# Benign follicular tumors*

**DOI:** 10.1590/abd1806-4841.20154114

**Published:** 2015

**Authors:** Oscar Tellechea, José Carlos Cardoso, José Pedro Reis, Leonor Ramos, Ana Rita Gameiro, Inês Coutinho, António Poiares Baptista

**Affiliations:** 1 Centro Hospitalar Universitário de Coimbra – Coimbra, Portugal.

**Keywords:** Birt-Hogg-Dube syndrome;, Hair follicle;, Hamartoma syndrome, multiple, Neoplasms, adnexal and skin appendage

## Abstract

Benign follicular tumors comprise a large and heterogeneous group of neoplasms that share a common histogenesis and display morphological features resembling one or several portions of the normal hair follicle, or recapitulate part of its embryological development. Most cases present it as clinically nondescript single lesions and essentially of dermatological relevance. Occasionally, however, these lesions be multiple and represent a cutaneous marker of complex syndromes associated with an increased risk of visceral neoplasms. In this article, the authors present the microscopic structure of the normal hair follicle as a basis to understand the type and level of differentiation of the various follicular tumors. The main clinicopathological features and differential diagnosis of benign follicular tumors are then discussed, including dilated pore of Winer, pilar sheath acanthoma, trichoadenoma, trichilemmoma, infundibuloma, proliferating trichilemmal cyst/tumor, trichoblastoma and its variants, pilomatricoma, trichodiscoma/fibrofolliculoma, neurofollicular hamartoma and trichofolliculoma. In addition, the main syndromes presenting with multiple follicular tumors are also discussed, namely Cowden, Birt-Hogg-Dubé, Rombo and Bazex-Dupré-Christol syndromes, as well as multiple tumors of follicular infundibulum (infundibulomatosis) and multiple trichoepitheliomas. Although the diagnosis of follicular tumors relies on histological examination, we highlight the importance of their knowledge for the clinician, especially when in presence of patients with multiple lesions that may be the cutaneous marker of a cancer-prone syndrome. The dermatologist is therefore in a privileged position to recognize these lesions, which is extremely important to provide further propedeutic, appropriate referral and genetic counseling for these patients.

## INTRODUCTION

Benign hair follicle tumors (BHFT) encompass a large number of relatively rare neoplasms defined by the type and degree of hair follicle differentiation as seen on their histologic examination.

They generally occur on the head and neck of adults as a nondescript slow-growing solitary papule or nodule, and are mostly of exclusive dermatologic relevance. However they can possess peculiar clinical features enabling the diagnosis and, most importantly, they may be the first clinical manifestation of complex visceral cancer-prone syndromes. In addition, some can mimic primary malignant skin neoplasms or possess a malignant counterpart from which they should be distinguished. Occasionally they can be misdiagnosed as benign or malignant sweat gland tumors.

Diagnostic criteria for BHFT are well established and, from a practical point of view, the main issue about these neoplasms concerns their differential diagnosis, i.e. the distinction among different BHFT depicting some type of hair follicle differentiation and the distinction between certain types of BHFT and basal cell carcinoma.^1,2^

Their pathogenesis remains largely unknown. Loss of heterozygosity of mutated tumor suppressor genes has been implicated in the genesis of some sporadic cases of BHFT in an analogous manner to their respective hereditary counterparts.^3^ Additionally, in some cases a relationship with viral infection has been suggested.^4^

As the precise diagnosis of these tumors, required by the reasons explained above, depends on the similarity of their histologic phenotype to the microscopic features of the normal hair follicle, a short review of the latter is provided emphasizing the distinctive characteristics of the different anatomical regions of the adnexal.

## MICROSCOPIC ASPECTS OF HAIR FOLLICLE DIFFERENTIATION

**Figure 1:**
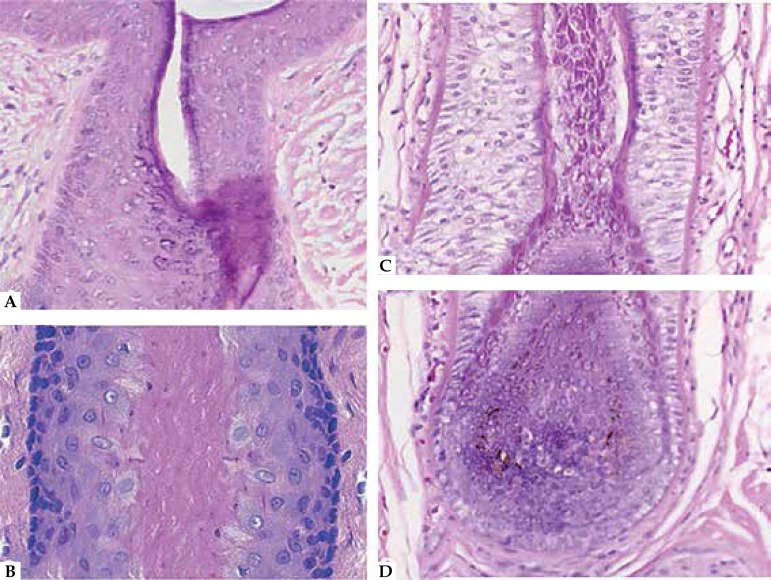
Different segments of the hair follicle. **A.** Infundibulum. Keratinization similar to the epidermis, including a granular layer and basket-weave orthokeratosis. **B.** Isthmus. Trichilemmal keratinization, characterized by absence of granular layer and abrupt compact orthokeratosis. **C.** Stem. Pale outer root sheath cells surrounded by a hyaline membrane. **D.** Bulb. Closely packed basophilic keratocytes and interspersed pigmented melanocytes characteristic of the hair matrix

The mature anagen hair follicle can be divided in different segments: infundibulum, isthmus, "stem" and bulb, distinguishable from each other by the organization and morphology of the cells that compose them, as well as their relative disposition (Figure 1). Beyond these regions that correspond to a vertical section of the hair follicle, two sheaths, resulting from a horizontal section of the hair follicle, can be also defined: i) an outer hair sheath (ORS) extending from the base of the bulb to the base of the infundibulum, which lays on a basement membrane that in some areas is thickened and hyaline (vitreous membrane); and ii) an inner root sheath (IRS) that is continuous along the bulb and the stem, disappearing at the isthmus. ORS is composed of pale cells that are similar along the entire ORS. In contrast, IRS is more complex as three concentric cell layers are identifiable: Henle layer, Huxley layer and cuticle. The more peripheral, Henle layer is a single row column that encloses the double row Huxley layer. These layers extend vertically from the bulb to the stem and are composed of cells characterized by the presence of conspicuous eosinophilic trichohyalin granules (the hair equivalent to keratohyalin). These cells undergo cornification near the level where the stem initiates, resulting in compact corneocytes characterized by their gray-blue hue. Cornification is seen earlier (before the beginning of the stem) in Henle layer than in Huxley layer. Cuticle is the most central layer of IRS and consists of axially oriented flat minute pale cells.

### Signs of infundibular differentiation

The infundibular epithelium is indistinguishable from that of the adjacent epidermis (Figure 1A), consisting in:

(i) a basal layer;

(ii) a spinous layer with conspicuous intercellular bridges;

(iii) a granular layer of granular cells with keratohyalin granules identical to those of the epidermis; and

(iv) a basket-weave stratum corneum. It should be noted that the infundibulum is not a constituent of the outer root sheath (ORS).

### Signs of Outer Root Sheath differentiation (isthmus; stem)

The ORS (trichilemma) consists of rows of keratinocytes with pale, abundant PAS-positive cytoplasm, which cornify horizontally and abruptly without a granular cell layer, originating the compact eosinophilic stratum corneum (Figures 1B and 1C). The thickness and hue of ORS changes along its length: thin and clear at the bulb, thicker and more eosinophilic (pink) at the stem, and again thinner at the isthmus. Although ORS cells are bound by desmosomes, inter-cellular bridges are seldom conspicuous.

Signs of ORS differentiation include:
(i) pale/clear, PAS-positive cells with no conspicuous intercellular bridges;
(ii) absence of granular layer; and
(iii) a compact eosinophilic stratum corneum.

Some phenotypic differences between the upper (isthmus) and lower (stem) segments of ORS exist (more striking nuclear palisading and pinker cytoplasm with higher content of glycogen of ORS cells in the stem).

Signs of Inner Root Sheath differentiation:
(i) Cells with conspicuous bright eosinophilic trichohyalin (at the bulb);
(ii) Compact blue-gray corneocytes (stem).

**Signs of differentiation to the bulb and papilla**
(i) Inverted cup disposition of matrical cells involved by
(ii) the lower (bulbar) segment of IRS, i.e. two to three columnar rows of cells with conspicuous bright eosinophilic tricohyalin, that is, in turn, surrounded by
(iii) the most inferior portion of ORS (a thin layer of pale PAS positive cells).

Signs of hair matrix differentiation (Figure 1D)
(i) Small cells with a scant basophilic cytoplasm and voluminous round nuclei with prominent nucleolus, occurring in crowded aggregates;
(ii) Mitotic figures;
(iii) Necrosis of isolated cells (karyorrhexis and picnosis);
(iv) Dendritic melanocytes (occasionally).

**Signs of hair shaft differentiation**
(i) Ghost cells (anucleated polygonal eosinophilic cells centered by an empty space replacing the nucleus);
(ii) Refractile yellow-orange cornified cells;
(iii) True hair shaft (rarely observable).

**Signs of differentiation towards the perifollicular sheath**
(i) Loosely arranged, thin collagen fibers in a mucinous matrix;
(ii) Scattered fibroblasts with round or oval nucleus;
(iii) Numerous capillary blood vessels.

#### Signs of differentiation towards the follicular germ

Crescent-shaped, oval or round aggregates of cells with oval nucleus, and scant, intensely basophilic cytoplasm (basaloid cells) with peripheral palisading, recapitulating the follicular hair germ.

## CLASSIFICATION

The classification of the benign follicular proliferations is histogenic and based on the set of microscopic similarities between lesions and normal hair follicle structures to which they can be contiguous and that are recapitulated at different degrees in the tumor (Chart 1).

**Chart 1:** Classification of benign follicular tumors

**Infundibular and/or isthmic differentiation**	
	Dilated pore (Winer)
	Pilar sheath acanthoma
	Pilar sheath acanthoma
	Trichilemmoma
	Desmoplastic trichilemmoma
	Infundibuloma (tumor of follicular infundibulum)
	Pilar tumor (proliferating trichilemmal cyst)
	
**Germinative cell differentiation**	
	Trichoblastoma
	Adamantinoid trichoblastoma
	Trichoepithelioma
	Trichogerminoma
	Panfolliculoma
	
**Matrical differentiation**	
	Pilomatricoma
	
**Mesenchimatous follicular differentiation**	
	Trichodiscoma/Fibrofolliculoma
	Neurofollicular hamartoma
	Perifollicular fibroma
	Trichofolliculoma
	Folliculosebaceous cystic hamartoma

### Dilated pore (Winer)

It occurs as a small solitary papule centered by a follicular pore on the face, neck or back, mimicking a giant comedo. It can be multiple and, exceptionally, it is agminated with a linear distribution (*dilated pore nevus*).^5^

Histologically it is a small vertically oriented infundibulocystic lesion, filled with lamellar ortokeratosis.^6^ The wall recapitulates the infundibular epithelium and contains regularly spaced elongated rete ridges, slightly thicker than those of the adjacent epidermis. These infundibular radiary crests do not contain keratinous microcysts or sebaceous ducts, as is the case of pilar sheath acanthoma (see below). In serial sections a subjacent infundibular cyst may be occasionally found.

Although it has been suggested that dilated pore of Winer merely corresponds to an infundibular cyst opening to the skin surface with reactive wall hyperplasia surrounded by fibrotic adventitial dermis, the identity of the lesion within the follicular tumours has been reaffirmed. ^7^

Differential diagnosis

Pilar sheath acanthoma: See below.

Comedo: It is an operculized dilated infundibular cavity filled with lamelar ortokeratosis and limited by infundibular epithelium with no elongated rete ridges.

### Pilar sheath acanthoma

It is a rarely reported diagnosis presenting clinically as a solitary papule centered by a keratotic plug on the upper lip or central area of the face.

**Figure 2:**
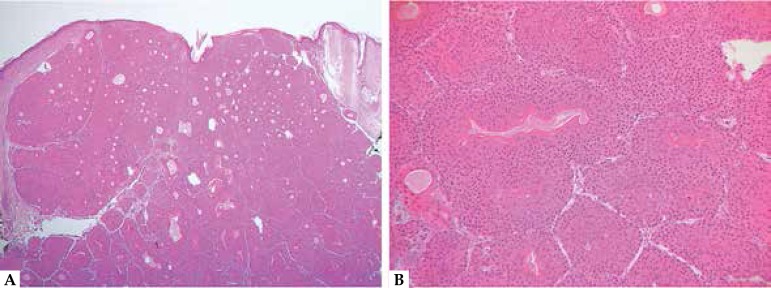
Pilar sheath acanthoma. A. This is a particularly large and solid example; note the multilobular architecture. B. Predominantly isthmic differentiation with abrupt compact keratinization

Microscopically, it is a well-circumscribed, vertically oriented epithelial cystic proliferation of the dermis, opening on the skin surface and filled by lamellar keratin.^8^ Peripheral lobules of infundibular or isthmic (pale, PAS+) cells irradiate from the acanthotic and hyperplastic cyst wall (Figure 2). Within the radiate lobules, where the cells of the most peripheral layer commonly display nuclear palisading, small horn cysts and, less frequently, sebaceous ducts, sebocytes or tiny squamous eddies may be seen.

Thus, the lesion is considered to be made of a more superficial infundibulocystic portion and a deeper lobulated and radiary region with isthmic differentiation.^1^

*Differential diagnosis*

*Dilated pore (Winer)*: The peripheral radial ridges of dilated pore are regular, evenly spaced and made of infundibular rather than isthmic keratinocytes and do not contain keratinous cysts. Distinction can be less straightforward in oblique sections.

*Trichofoliculoma*: As pilar sheath acanthoma, trichofolliculoma has a vertically oriented well-circumscribed infundibulocystic silhouette. However, smaller infundibula rather than pale cell lobules irradiate from the main wall, resulting in minute dystrophic or more mature vellus hairs, features that contrast with the less florid pattern of pilar sheath acanthoma.

Due to the occasional extension to deep dermis/hypodermis and to the radiate projections, the treatment of choice is surgical excision with a 2 mm margin from the ostium.

### Trichoadenoma (Nikolowski)

It is a rare lesion occurring in adults with no gender predilection, presenting as a 3-15 mm nondescript papule/nodule on the face, neck or trunk.

Histologically it corresponds to a well-circumscribed, horizontally oriented, dermal nodule, with no continuity with the overlying epidermis. The tumor is composed of numerous, relatively large, round or oval, infundibulocystic structures, separated by a poorly developed fibrous stroma (proportion epithelium/stroma: “10:1”). The epithelial cysts are occasionally contiguous, and solid cords projecting from the thin cystic wall, which may appear “isolated” in the stroma, can occur. Calcifications are typically absent.^9,10^

#### Differential diagnosis:

*Trichoepithelioma*: As trichoadenoma, it is a horizontal intradermal well-defined tumor composed of keratinous infundibulocysts depicting granular layer. However, nests of germinative cells in nodular, trabecular and cribriform arrays coexist in different proportions. Also, contrasting with trichoadenoma, follicular germ, papillar or bulb differentiation and retraction clefts within the stroma are characteristically seen in trichoepithelioma.

*Desmoplastic trichoepithelioma*: It displays a general picture similar to trichoadenoma but possesses a characteristic central depression and a much more developed and denser stroma. In addition, most cells have features of germ cells rather than infundibular differentiation, and calcification is often seen, a feature characteristically absent in trichoadenoma.

*Trichilemmal cyst nevus*: Although at low magnification *trichilemmal nevus* may evoke the diagnosis of trichoadenoma, the former consists of isthmic structures, with no granular layer ("agminated trichilemmal cysts"), contrasting with the infundibular differentiation phenotype of trichoadenoma.^11^

*Microcystic adnexal carcinoma*: Can be distinguished from trichoadenoma by its irregular, asymmetrical silhouette, deep extension, abundant scirrhous stroma and characteristic perineural invasion.

### Tricholemmoma

Two types of clinical presentation of trichilemomma are recognized.

The more common type occurs as a solitary papule on the face of adults, generally misdiagnosed as a verrucous papilloma, a viral wart or a basal cell carcinoma. Rarely does it correspond to a cutaneous horn.^12^

In exceptional cases the patient presents, typically after the second decade of life, with multiple papules of the face (nasolabial folds, upper lip, front, ears). In this variant, cutaneous lesions (trichilemommas) coexist with small papules on the oral cavity (gums, tongue), which tend to coalesce in a cobblestone pattern corresponding to fibromas. This "florid" clinical picture constitutes the classic mucocutaneous presentation of Cowden's syndrome, which shows multiple extracutaneous neoplasms (breast, kidney, intestine) and depends on germline mutations of the tumour suppressor gene PTEN. It is generally admitted that the presence of 3 or more histologically proven trichilemommas constitutes a major criteria for diagnosis of Cowden's syndrome. ^13^ However, more recently, in the new proposed diagnostic criteria, the presence of at least 3 trichilemmomas, one of which must be biopsy-proven, is considered enough to fulfil one major criterion. ^14^

It is likely that other entities characterized by similar germline PTEN mutations including: (i) Lhermitte-Duclos disease (hamartomatous cerebellar dysplastic gangliocytoma); (ii) Bannayan Riley Ruvacalba syndrome (pediatric hamartomatous syndrome displaying multiple subcutaneous lipomas, hemangiomas, intestinal polyps and macrocefaly); and (iii) segmentar forms of a Proteus-like syndrome, constitute, with the Cowden's syndrome, poles of a symptomatic spectrum depending of germline PTEN mutations and generically designated as PTEN Hamartoma Tumor Syndrome (PHTS).^15,16^

**Figure 3:**
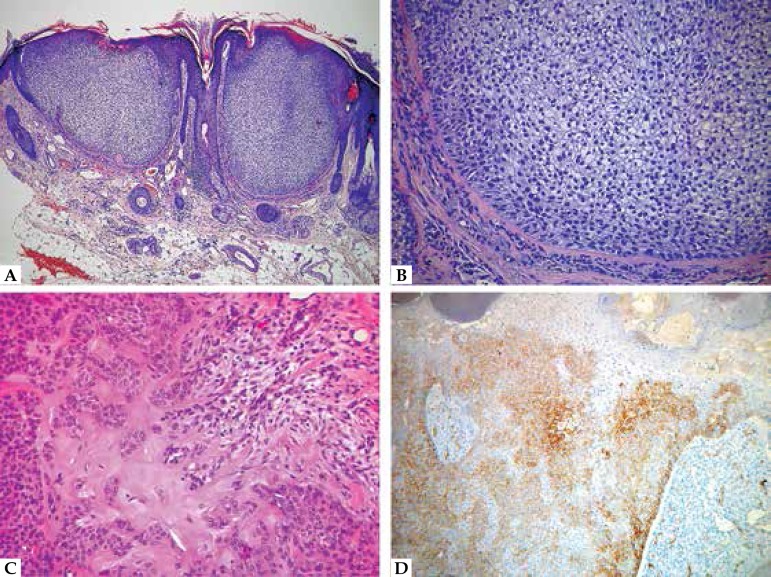
Trichilemmoma. A. Typical lobular architecture in close relation with the neighboring hair follicles. B. Detail of the pale cells with a hint of peripheral palisading and surrounding hyaline membrane. C. Detail of a desmoplastic trichilemmoma, with sclerotic stroma and apparently infiltrative architecture. D. CD34 immunohistochemistry, demonstrating positivity in the tumor cells

Microscopically the aspect is identical in solitary and Cowden's associated variants: a folliculocentric lobular proliferation of polygonal, clear, PAS-positive isthmic cells with nuclear palisading of the peripheral cells that characteristically lay on a thickened hyaline eosinophilic basement membrane (Figures 3A and 3B). Squamous eddies are frequently seen, occasionally centered by foci of infundibular keratinization. Pigmentation, necrosis or calcification may occur. Rarely the overlying epidermis is hyperplastic.

Some cases of solitary trichilemmoma (as well as desmoplastic trichilemomma, see below) could represent a reorganized viral papilloma, as testified by the occasional presence in these cases of signs of cytopathogenic HPV effect. However this interpretation, as well as the molecular finding of HPV in trichilemmomas, remains controversial.^17,18^

In Cowden's syndrome, beyond trichilemmomas, follicular inverted keratosis, infundibular hyperplasia, or tumor of the follicular infundibulum (see below) have been reported. In addition, acrokeratosis verruciformis-like lesions can occur. Oral fibromas of Cowden's syndrome are indistinguishable microscopically from the common oral fibroma.

#### Differential diagnosis

*Infundibuloma*: As trichilemmoma, it is made of infundibular cells, larger and paler than the peripheral basaloid cells, displaying nuclear palisading and laying at the dermal border of the lesion. However, in contrast to trichilemmoma, infundibuloma has a subepidermal plaque-like general arrangement and no peripheral hyaline membrane, but instead, a characteristic reinforcement of the elastic tissue.

*Epidermal panfolliculoma*: This acanthotic intraepidermal follicular proliferation, although containing areas of trichilemmal differentiation, recapitulates both upper and lower hair follicle segments exhibiting, in addition, germinative, matrix, bulb, papilla, and inner root sheath microscopic phenotypical features.

*Other intraepidermal clear cell neoplasms. Clear cell acanthoma*: lower limb localization, well circumscribed acanthotic lesion of pale and large keratinocytes covered with a psoriasiform scale, distinct demarcation from intraepidermal adnexal epithelium and surrounding keratinocytes. *Clear cell poroma*: absence of both peripheral palisading and hyaline peripheral membrane, sharp contrast with neighboring epidermis, dermal extension, ductal differentiation. *Clear cell squamous cell carcinoma in situ*: nuclear atypia, absence of basement membrane.^19^

### Desmoplastic trichilemmoma (DT)

DT constitutes a type of solitary tricholemmoma characterized by a pseudo invasive silhouette imparted by the disposition of the stroma that dissects the epithelial tumor lobules (Figure 3C). Peripheral palisading of nuclei may be absent.^20,21^

#### Differential diagnosis

*Basal cell carcinoma*: In DT no clefts between the epithelium and stroma, nor cellular atypia or apoptosis, are found. In superficial biopsies the distinction may be less obvious and in these cases the differential expression of BerEP4 (present in CBC, absent in DT) and CD34 (absent in CBC, present in DT - Figure 3D) may be helpful.

**Tumor of follicular infundibulum (infundibuloma)**

**Figure 4:**
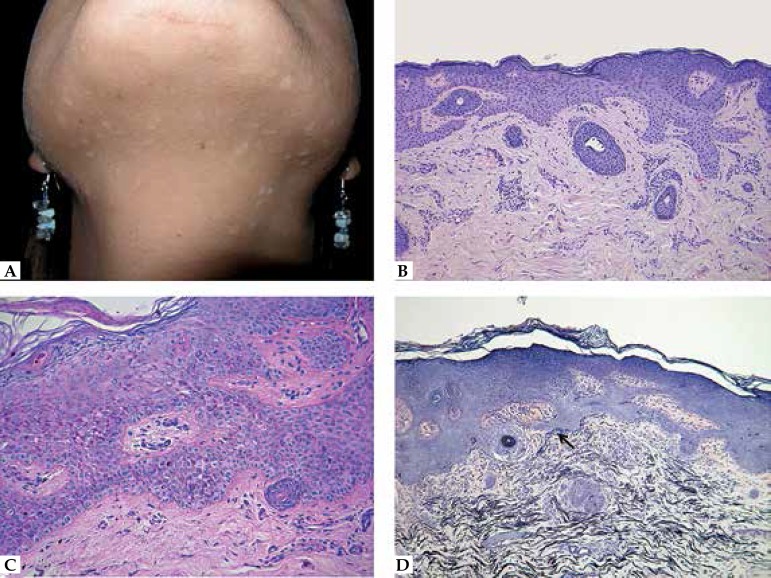
Multiple tumors of follicular infundibulum (infundibulomatosis). A. Multiple hypopigmented macular or slightly papular lesions in the submandibular region. B. Plate-like proliferation of monomorphous pale keratinocytes, well-demarcated from the adjacent epidermis. C. PAS stain is positive in the cells. D. Condensation of elastic fibers around the base of the proliferation (arrow)

Two clinical variants occur.^22,23^ More commonly the lesion is a solitary keratotic papule of the face, neck or upper trunk in elderly patients, judged as a seborrheic keratosis or basal cell carcinoma. Exceptionally, multiple (<20 to >100) lenticular, hypopigmented or skin-colored, maculopapular or slightly atrophic elements are seen on the face, neck and upper trunk of young adults, simulating plane warts, pityriasis versicolor, guttate hypomelanosis or vitiligo (Figure 4A).^24^ Atrophic lesions may be confused as acne scars and erythematous ones may resemble actinic porokeratosis. Although the lesions are benign, the possibility of "transformation" into basal cell carcinoma as well as its occurrence within the spectrum of the cutaneous lesions of Cowden syndrome have been described, making follow-up of these patients advisable. ^25,26^ It can also arise within nevus sebaceous.

Histologically, the tumor consists of a well circumscribed, subepidermal, horizontal, plate-like proliferation of pale-staining, PAS-positive keratinocytes (Figures 4B and 4C). Cells are monomorphous with no atypia; peripheral palisading of nuclei is a common feature. The peripheral cells may lie on a thickened basement membrane, and small keratinous pseudocysts may occur. A unique feature, peculiar to the tumor of follicular infundibulum, is the presence of a network of elastic fibers surrounding the lower margin of the tumor (Figure 4D).

Immunohistochemically the lesion stains for MNF-116 and CK5/6, and is negative for Ber-EP4, Bcl2, CK7 and CK20.

##### Differential diagnosis:

*Superficial basal cell carcinoma*: Pale cells are seldom prominent; the pattern is "multicentric" in nests, rather than in plaque; clefts are obvious between the epithelium and the underlying stroma; tumor cells are frequently atypical, and show BerEP4 and Bcl2 immunoreactivity, features that sharply contrast with the findings in infundibuloma.^24,27^

Trichilemmoma: Infundibuloma lacks the lobular folliculocentric pattern, and peripheral hyaline basement membrane typical of trichilemomma, depicting a subepidermal anastomosing plaque-like pattern and reinforcement of the peripheral elastic tissue, features not found in trichilemmoma.

*Epidermal panfolliculoma*: infundibular plate like areas made of pale cells may occur, but only focally, the lesion being characterized by a recapitulation of the entire spectrum of hair follicle.

It should be noted that cases described as "*Multiple infundibular tumors of the head and neck*" bear no resemblance with infundibulomatosis and exhibit histological features akin to those of prurigo nodularis.^28^

The putative relationship between infundibuloma and basal cell carcinoma (BCC) as well as the eventuality of "transformation" of infundibuloma into BCC, although controversial, may justify the removal of solitary lesions.^29^ In infundibulomatosis, cryotherapy, CO_2_ laser, topical retinoids, or keratolytics may be attempted.

#### Pilar tumor (proliferating trichilemmal cyst)

Typically pilar tumor (PT) is a large (2-25 cm), exophytic nodule/tumor with a smooth, often ulcerated surface, present for years on the scalp of elderly women, and clinically interpreted as a squamous cell carcinoma.^30^ It may occur *de novo* or arise on a pre-existing trichilemmal cyst, and it may have an extra-cephalic localization. Recurrence is rare and metastases occur exceptionally.^31^

**Figure 5:**
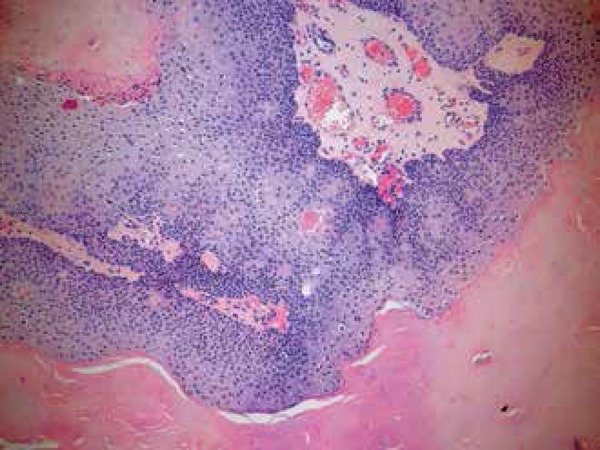
Proliferating trichilemmal cyst/tumor. Partially cystic and solid lesion with keratinocytes increasing in size from the basal layer to the lumen, displaying abrupt keratinization without granular layer

The diagnosis requires the identification, at least focally, of a trichilemmal cyst and the presence of significant cytological atypia. It resides on the dermis and/or hypodermis and may be contiguous with the hair follicle. It corresponds to a well-circumscribed, large, rounded solid/cystic tumor with a smooth border surrounded by a dense pseudocapsule that is separated by clefts from the adjacent tissue. Irregular anastomosing strands of keratinocytes irradiate centripetally from the tumor wall. These strands are successively composed of: i) basal layer; ii) cells with abundant eosinophilic cytoplasm similar to the outer root sheath at the isthmus; and iii) a horny layer in direct contact with the isthmic cells, without interposition of a granular layer (Figure 5). These cornified foci occur frequently as scattered islands surrounded by the isthmic cells. Mitotic figures as well as dyskeratotic keratinocytes may be observed and cytological atypia is characteristically prominent. Calcification is common. The epithelial strands may lie on a peripheral hyaline thickened basement membrane.

##### Differential diagnosis:

The intensity of cytologic atypia and the presence of mitotic figures and dysketatosis in an epithelial tumor of eosinophilic cells that is only partially cystic may arise the suspicion of squamous cell carcinoma. However, the asymmetric, irregular and invasive border, focal contiguity/ replacement of the epidermis of the latter contrast with the low and higher magnification features of PT that additionally exhibits "trichilemmal" rather than epidermal cornification.

Despite the intensity of the atypical cytological features, PT behaves most often in a benign fashion. The rarities of regional lymph node metastases as well as the local symptoms warrant a complete surgical excision.

#### Trichoblastoma

It is a benign neoplasm made of germinative hair follicle cells with a differentiation generally restricted towards the hair germ and papilla. It is relatively infrequent except in the setting of organoid nevus. Clinically it presents as a dermal or dermo-hypodermal nodule of the head or neck. When it occurs within an organoid nevus it may be clinically felt to be a BCC.

Several forms of trichoblastoma have been reported including giant, subcutaneous, pigmented and clear cell variants. "Cutaneous lymphadenoma" is generally considered an adamantinoid variant of trichoblastoma (see below). Also, trichoepithelioma is thought to correspond to a trichoblastoma with advanced differentiation (see below).

Typically it corresponds to a dermal and/or hypodermal, well circumscribed, symmetrical tumor with no contiguity with the surface epithelium, composed of irregular nests of small basophil cells, resembling a BCC. Within the tumor the cellular aggregates exhibit different patterns: nodular, adenoid, cystic, and trabecular. The latter can be cribriform, racemous, reticulated or "schwannoid" ("*rippled*").^1^

Two cell types constitute the cell aggregates of trichoblastoma: i) basaloid cells, with inconspicuous cytoplasm and appearing to contain solely a basophil nucleus (similar to follicular germinative cells); ii) cells with larger, pink cytoplasm alike hair stem cells.

In most instances there is a strong predominance of the germinative (basaloid) cells. However the relative proportion of the 2 cell types is somewhat related to the architectural pattern of the cell aggregates (e.g.: essentially germinative cells in the nodular pattern; equivalence of germinative and stem cells in the reticulated pattern). Hair papilla and hair germ formation ("limited hair follicle differentiation") is typically found in trichoblastoma.

**Figure 6:**
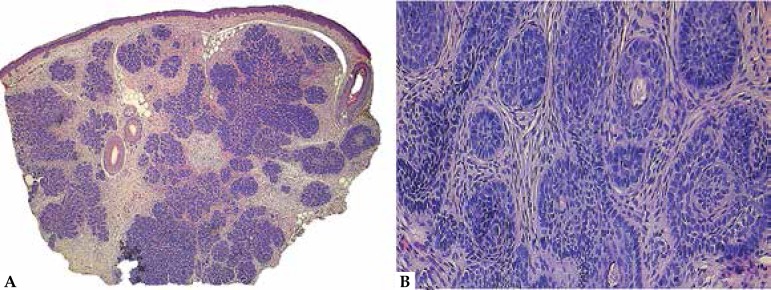
Trichoblastoma. A. Basaloid proliferation with dense stroma separated by clefts from the adjacent dermis. B. Detail of the cellular stroma forming condensations near the basaloid islands (papillary mesenchymal bodies)

Surrounding the epithelial aggregates a conspicuous, cellular or dense stroma is found, which characteristically contains clefts present between the stroma and the surrounding dermis, but usually not between the stroma and the epithelial aggregates (Figure 6).

Melanin in the cell nests is not rare, namely in trichoblastomas associated with organoid nevi. Despite some cellular pleomorphism, no cytologic atypia or mitoses are found. Similarly, pronounced tumoral necrosis is absent, but individual apoptotic bodies with occasional confluence may be observed.

Frequently, continuity with the follicular infundibulum is seen, and infundibulocystic differentiation may occur within the nests of eosinophilic cells. When the latter is prominent and the lesion is superficial, the histologic picture is indistinguishable from that of trichoepithelioma (see below).

##### Differential diagnosis:

The most common difficulty in the differential diagnosis is to distinguish trichoblastoma from BCC. In most instances the asymmetric silhouette, cytologic atypia, occasional mitoses, presence of clefts between the cellular nests and the stroma with its mucinous character, allow the diagnosis of the latter. In partial or superficial biopsies, and occasionally in particular cases, the distinction may be more difficult. When the whole lesion is not available for microscopic examination and if immunohistochemistry is performed in these cases, CK15 immunoreactivity of the cellular aggregates as well as the presence of scattered CK20-positive cells would favour the diagnosis of trichoblastoma. In the same way, the differential expression of CD10 and Bcl2 (CD10 positive on the stroma of trichoblastoma, but not on BCC cells; and Bcl2 immunoreactivity positive in BCC cells, but negative in trichoblastoma cells) may be helpful in the distinction.^32,33^

Trichoepithelioma would correspond to a superficial variant of trichoblastoma with prominent infundibulocystic differentiation (see below).

Trichoblastoma is a benign tumor and the complete surgical excision (often necessary for a correct histologic diagnosis) is the preferable treatment modality. Rarely it can have malignant evolution secondary to the sarcomatous transformation of the stroma or coexist with other malignant adnexal neoplasm(s) within an organoid nevus.^34^

#### Adamantinoid trichoblastoma (Lymphoepitelial cutaneous tumour; cutaneous lymphadenoma)

It is an uncommon neoplasm with differentiation towards the hair germ characterized by the permeation of the tumoral tissue by a conspicuous lymphocytic infiltrate, a feature reflected on the initial designations of "lymphoepitelial cutaneous tumour" and "cutaneous lymphadenoma". Currently the tumor is considered a variant of trichoblastoma.

Clinically, it corresponds to a slowly growing solitary non-descript nodule on the face or lower limb of adults. Pediatric cases are exceptional.

**Figure 7:**
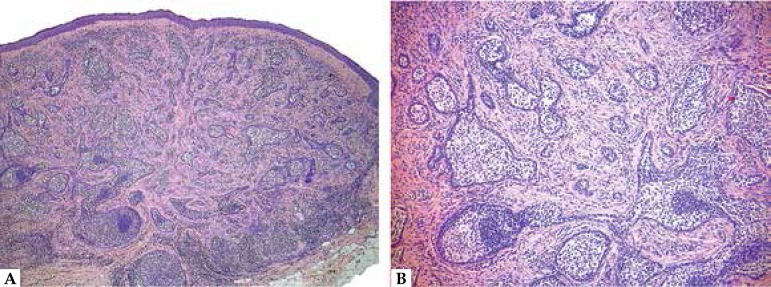
Trichoblastoma. A. Trichoblastoma. A. Basaloid proliferation with dense stroma separated by clefts from the adjacent dermis. B. Detail of the cellular stroma forming condensations near the basaloid islands (papillary mesenchymal bodies)

Microscopic examination generally discloses a well-circumscribed dermal tumor made of epithelial nests and strands, variable in size and shape, in which two cell types are readily identifiable: (i) basophilic, germinative cells with a tendency for nuclear palisading at the periphery of the epithelial aggregations; and (ii) loosely arranged cells with more prominent and paler cytoplasm occupying the centre of the tumor nests. Typically, small mature lymphocytes conspicuously infiltrate the tumor epithelium. As a rule the epithelial aggregations are embedded in a dense stroma (Figure 7).^35,36^

The (i) cytoarchitectural compartmentalization of the tumor nests, with basaloid peripheral cells and loosely arranged pale, glycogen-rich central cells, observable from low magnification; (ii) the lymphoid cell infiltration; and (iii) the presence of scattered S100 and CD1a-positive dendritic cells, are highly characteristic and distinctive of adamantinoid trichoblastoma, which seldom poses problems in terms of differential diagnosis.

Distinction from trichogerminoma, another hair germ tumor considered by some as another variant of trichoblastoma (see below), is based on the typical concentric arrangement of the cells ("cell balls") on the centre of the tumor nests in trichogerminoma, which, in addition, lacks the characteristic lymphocytic cell permeation of adamantinoid trichoblastoma.

#### Trichoepithelioma

**Tricoepithelioma is thought to correspond to a superficial (intradermal) trichoblastoma with prominent infundibulocystic differentiation.**

Besides the more common solitary, clinically nondescript, sporadic type occurring in adults, lesions may be multiple, presenting in childhood or adolescence with autosomal dominant inheritance (epithelioma adenoides cysticum - OMIM 601606). In the latter type the patient presents with multiple 2-10 mm papules occasionally coalescing on the face (nasolabial folds) or scalp and neck. Linear blaschkoid distribution may occur. Coexistence with cylindromas or less frequently spiradenoma has been reported, in the context of CYLD gene mutations.^37^ Multiple trichoepitheliomas are, in addition, characteristic of Rombo syndrome and Bazex-Dupré-Christol syndrome (BDCS). In both of these syndromes trichoepitheliomas coexist with atrophoderma vermiculatum, milia, hypotrichosis and basal cell carcinoma. Distinction between them depends on i): localization of the atrophodermic changes: malar regions and elbows in Rombo syndrome versus dorsal hands in BDCS; ii) cyanotic erythema in Rombo syndrome present from infancy, not reported in BDCS, and: iii) X-linked transmission in BDCS contrasting to the autosomal dominant pattern of inheritance of Rombo syndrome.^38^

Occasionally giant solitary trichoepitheliomas of the thigh, buttock and perianal regions have been reported. More frequently the solitary forms are clinically misdiagnosed as a melanocytic nevus or a non-ulcerated BCC.

**Figure 8:**
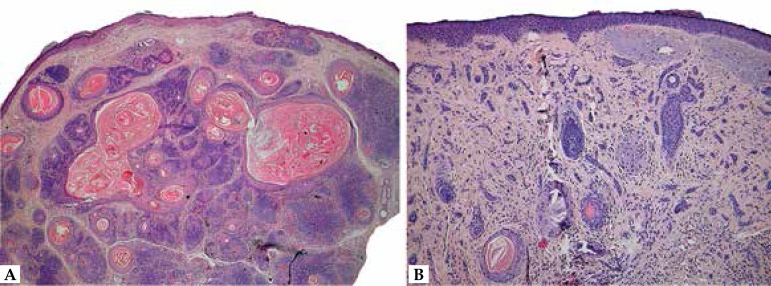
A. Trichoepithelioma. Basaloid proliferation with abundant infundibular keratinization; note the dense stroma separated by clefts from the adjacent dermis. B. Desmoplastic trichoepithelioma. Thin basaloid strands in a dense stroma, and a few A B keratocysts

Histologically, trichopithelioma is a well-demarcated intradermal tumor with a horizontal long axis separated from the neighbouring dermis by retraction clefts. The tumor is composed of nests of basaloid (hair germ) cells in a nodular or trabecular array coexisting with infundibulocystic structures (Figure 8A). In the latter, calcification may be seen. In the basaloid nests, hair germ/papilla and bulbar differentiation are present. Although peripheral nuclear palisading and individual cell necrosis may occur, no significant atypia, massive necrosis or atypical mitoses are seen. The stroma is loosely collagenous and contains elongated fibroblasts occasionally organized in small aggregates at the vicinity of the basaloid cell nests (papillary mesenchymal bodies, traducing hair papilla differentiation). With the exception of foreign body granulomatous reaction to ruptured keratocysts, inflammation is characteristically absent.

##### Differential diagnosis:

*Basal cell carcinoma (infundibulocystic)*. The cellular, fibrocytic nature of the stroma, the presence of clefts within the stroma and between the stroma and surrounding dermis, as well as the larger follicular differentiation spectrum (infundibular and germinative, papillary or bulb), all argue for the diagnosis of trichoepithelioma.

*Trichoadenoma*: as opposed to tricoepithelioma, trichoadenoma (i) is an strictly infundibular (and not germinative) hair tumor, (ii) displays dilated contiguous infundibular structures, and (iii) a scanty stroma (epithelium to stroma ratio of "10/1") (iv) that is devoid of clefts.

*Trichoblastoma*: For some authorities trichoepithelioma is a superficial trichoblastoma with more advanced (infundibular) differentiation (see above).

#### Desmoplastic trichopithelioma

Described by Brownstein and Shapiro, desmoplastic trichopithelioma is a variant of solitary trichoepitelioma, recognizable for its clinicopathologic singularities.^39^

Clinically it is a small annular plaque with a characteristic depressed centre and raised border occasionally presenting scattered tiny milia grains. It can mimic the clinical image of granuloma annulare. In most cases it occurs on the malar region or forehead of adult women. Exceptionally lesions are multiple, familial or non-familial, occurring as typical annular plaques on the face and neck.

Histologically it corresponds to a well-circumscribed, symmetrical, discoid lesion on the superficial and mid dermis with a characteristic central depression that can evoke the diagnosis at low magnification. The tumor is made of branching or more linear cords of basaloid cells and keratinous infundibulocystic structures (Figure 8B). The cords are irregular, giving rise to bizarre patterns, occasionally one cell thick. The keratinous cysts are frequently calcified and can exhibit centrifugal "tadpole" projections of their wall. The epithelial aggregations are embedded in a conspicuous compact and hypocellular, desmoplastic stroma. No cytologic atypia or atypical mitoses occur. Focal foreign body granulomatous inflammatory infiltrate can occur surrounding ruptured keratinous cysts.

##### Differential diagnosis

*Basal cell carcinoma*: in superficial (shave) biopsies histological distinction between trichoepithelioma and BCC may be vexing; the localization of clefting within the stroma, or between stroma and neighbouring dermis, rather than between stroma and epithelial aggregates; the more extensive infundibulocystic differentiation; the more superficial and symmetric silhouette; as well as the absence of necrosis, mitosis and of inflammatory infiltrate, argue in favour of DT. Additionally, in contrast with morpheaform BCC, CK20/Cam 5.2-positive cells are often seen within the epithelial aggregates of DT.^2^

*Syringoma*: Syringoma may show keratinous cysts with wall rupture and foreign body reaction, and be misdiagnosed as DT and *vice versa*. However, syringoma is more superficially located than DT, exhibits ductal differentiation, and does not show germinative cells or clefting within the stroma. One cell thick strands or cords are not a feature of syringoma. EMA and CEA regularly decorate the luminal border and more occasionally the luminal cells of syringoma, but are not expressed in DT.

*Microcystic adnexal carcinoma (MAC)*: the characteristic features of MAC that allow its distinction from DT (deep extension, infiltrative pattern, asymmetry and perineural invasion) are not always present in small/superficial/shave biopsies, in which the distinction between these two neoplasms may not be possible. Immunohistochemical data described in MAC that can assist the differential diagnosis include expression of CααSMA) of the peripheral cells of ductal structures, and absence of BerEP4 and CK20/Cam5.2 immunoreactivity in tumor strands.

#### Trichogerminoma

Trichogerminoma is an insufficiently known benign follicular neoplasm described by Sau et al in 1992.^40^

It is a germinative hair follicle tumor with an intermediate differentiation between trichoblastoma and panfolliculoma (Table 1). From a clinical standpoint it is a nondescript papule or nodule of the head or neck.

**Figure 9:**
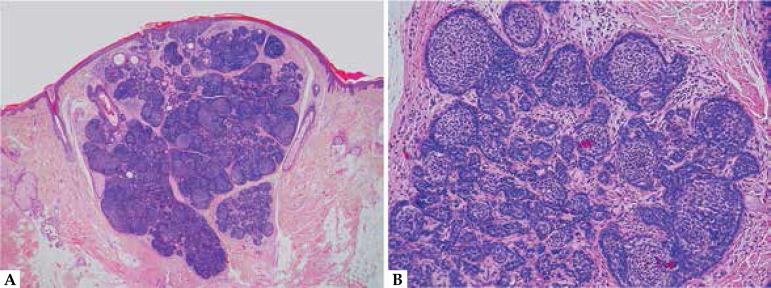
Trichogerminoma. A. Well-circumscribed intradermal multinodular lesion separated from the adjacent dermis by clefts. B. Detail of the “cell balls” composed of concentrically arranged pale cells surrounded by an outer layer of darker cells

Microscopic examination discloses an intradermal symmetrical nodule composed by basophilic lobules within a fibrotic stroma well demarcated from the surrounding dermis by clefts (Figure 9A). Within the tumor lobules, rounded cell nests ("cell balls") made of concentrically arranged pale cells, are characteristically seen (Figure 9B). The pale cell nests are surrounded by one or more layers of small basaloid, germinative cells displaying nuclear palisading at the periphery of the lobules. Hair germ and bulb structures are routinely found. Infundibulocystic, isthmic structures, sebaceous ducts and sebocytes often coexist. Immunohistochemically a "zonal" pattern produced by expression of CK5/6, CK 5/8 e CAM 5.2 on the peripheral germinative cells contrasting with the absence of staining at the cell balls is typical and may be helpful in distinguishing trichogerminoma from the more classic nodular types of trichoblastoma. ^41,42^

In contrast with adamantinoid trichoblastoma (that also shows cytoarchitectural compartmentalization of the cell aggregates in a peripheral germinative cell and a central pale cell area), no lymphocytes or S100/CD1a-positive dendritic cells are found in trichogerminoma.

Trichogerminoma is thought to be a variant of trichoblastoma with specific histological ("cell balls") and immunohistochemical features.

#### Panfolliculoma

In 1993 Ackerman *et al*.^1^ described 6 cases of a benign hair follicle neoplasm akin to trichoblastoma with a nodular/cystic pattern but characterized by phenotypic microscopic features of bulbar, matrical, stem, isthmic and infundibular differentiation. As the tumor recapitulated all the epithelial components of the mature hair follicle the term panfolliculoma was coined. Panfolliculoma would represent a trichoblastoma depicting the most wide differentiation spectrum. Cystic and intraepidermal variants may occur ("epidermal panfolliculoma").^43,44^ In the latter, histologic examination discloses an epidermal acanthoma where features of differentiation for upper and lower hair follicle, including germinative cells, matrical cells, outer root sheath cells, trichohyalin granules indicative of inner root sheath differentiation, ghost cells and laminated basket wave cornification, coexist.

#### Pilomatricoma

**Figure 10:**
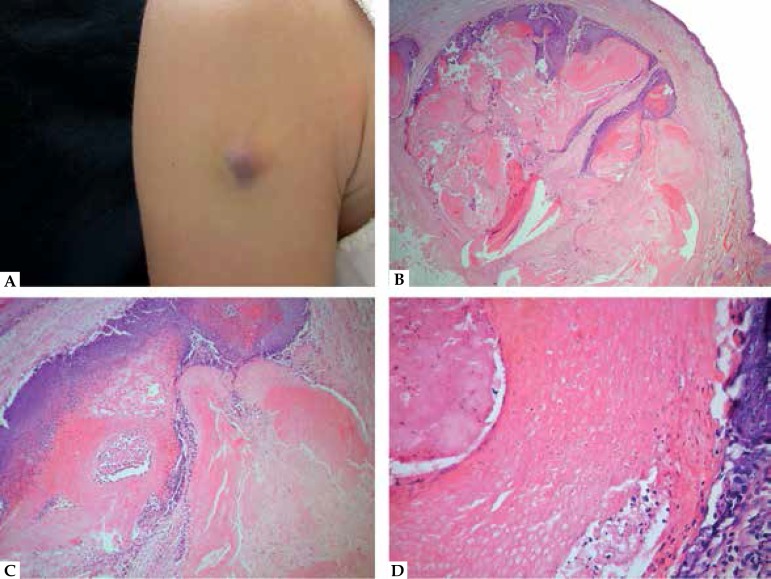
Pilomatrixoma. A. Nodule on the upper arm of a young patient; lesions are typically hard upon palpation, and a bluish color, as seen in this example, is not a rare feature. B. Well circumscribed dermal nodule with a biphasic appearance (basaloid and eosinophilic areas). C. Sharp transition between basaloid cells and keratinized strands. D. Detail of ghost cells, in which only the outline of the nuclei and cytoplasmic borders are discernible

Not infrequent in pediatric ages, where it constitutes the most common adnexal skin tumor, it presents clinically as a dermohypodermal progressively growing nodule of the head, neck, upper limbs and, less frequently, trunk or lower limbs (Figure 10A). Characteristically firm to hard on palpation, its consistency depends on the presence and extent of calcification. It may grow abruptly attaining large size mimicking a malignant lesion. In rare occasions it may be multiple, occurring in the setting of myotonic muscular dystrophy or of Gardner syndrome (familiar adenomatous polyposis) associated with high incidence of colorectal malignancy. More rarely, it can be the cutaneous marker of Rubinstein-Taybi syndrome or of chromossome 9 trisomy. ^45,46^ Occasionally the solitary lesions involute with anetodermic changes of overlying skin, suffer transepidermal elimination or are spontaneously painful.

β-catenin gene mutations have been described in most cases studied, implicating the WNT/β-catenin pathway in the pathogenesis of pilomatricoma, a feature in agreement with the association with Gardner´s syndrome, as this is characterized by germline mutations of a suppressor allele of this tumor suppressor pathway.^47^ More recently, chromosome 18 trisomy has been identified in pilomatricoma cells, a genetic anomaly that could additionally be pathogenically relevant via the hyperfunction of the anti-apoptotic gene Bcl-2, that resides in this chromosome.^48^

The histological picture varies with the age of lesions. Typically, it is a well-circumscribed dermohypodermal rounded nodule, with smooth borders surrounded by a pseudocapsule and constituted by epithelial aggregates (Figure 10B and 10C). In these aggregates two cellular types are regularly found: i) peripherally, cells are small with scant cytoplasm and a deeply basophilic elongated nucleus (matrical cells); they occur in multiple layers with no nuclear palisading; ii) in the centre of the epithelial aggregates, cells display an abundant eosinophilic cytoplasm whereas the nucleus disappears and is replaced by an empty space. These are known as ghost cells, they are characteristic of pilomatricoma and correspond to terminal differentiation of the basophilic peripheral cells (matrical cells) (Figure 10D). The transition between matrical cells and ghost cells may be abrupt or gradual, through a variable number of cells whose cytoplasm acquires progressive eosinophilia whereas the nucleus suffers pycnosis and is finally lost (Figure 10C).

In early lesions the architectural pattern is cystic and there is predominance of basophilic matrical cell aggregates. Progressively ghost cells replace the basophilic cells, and calcification ("calcified epithelioma") or, less often, hemosiderin deposition or even ossification, may occur. Simultaneously, a granulomatous foreign body inflammatory infiltrate is trigged, progressively replacing the epithelial proliferation. This aspect may dominate the histological picture in late lesions. Melanin granules and dendritic melanocytes may occur within the epithelial aggregates, reinforcing the phenotypic similitude between pilomatricoma and hair matrix. In some instances, particularly in older patients, the predominance of basophilic cells may persist in late lesions, a feature that frequently coexists with conspicuous mitosis ("proliferative pilomatricoma"). The histological picture is so characteristic that differentiation from other tumors is rarely put forward. When the basophilic cells are prominent the lesion can be misinterpreted as a "proliferative pilomatricoma" or even a malignant pilomatricoma. "Proliferative pilomatricoma" exhibits brisk mitotic activity, and predominance of matrical basophilic cells over ghost cells, but conserves the well circumscribed smooth border seen in ordinary pilomatricoma and should not be confused with the malignant pilomatricoma that, besides cytological atypia, variable mitotic rate and predominance of matrical cells, displays an asymmetrical silhouette, with poor circumscribed infiltrative jagged borders, central necrosis of the epithelial aggregates, and a lymphohistiocytic inflammatory infiltrate on a desmoplastic stroma.^49^ In partial superficial biopsies confusion with basal cell carcinoma may occur, but nuclear peripheral palisading and clefting between an altered stroma and the epithelial aggregates do not occur in pilomatricoma.

Pilomatricoma is a benign tumor. Nevertheless, locally aggressive and even metastatic examples have been rarely reported. Accordingly, surgical excision is advisable.

#### Trichodiscoma and Fibrofolliculoma

These two follicular neoplasms, characterized by extensive perifollicular mesenchymatous differentiation, although exhibiting distinct histologic features, generally coexist in the same patient, occasionally in identical nearby clinical lesions or in the same lesion (where both pictures can be seen in the same section or in serial sections) and are thought to correspond to different designations of the same entity with different development and topographic orientation of their constituents.

**Figure 11:**
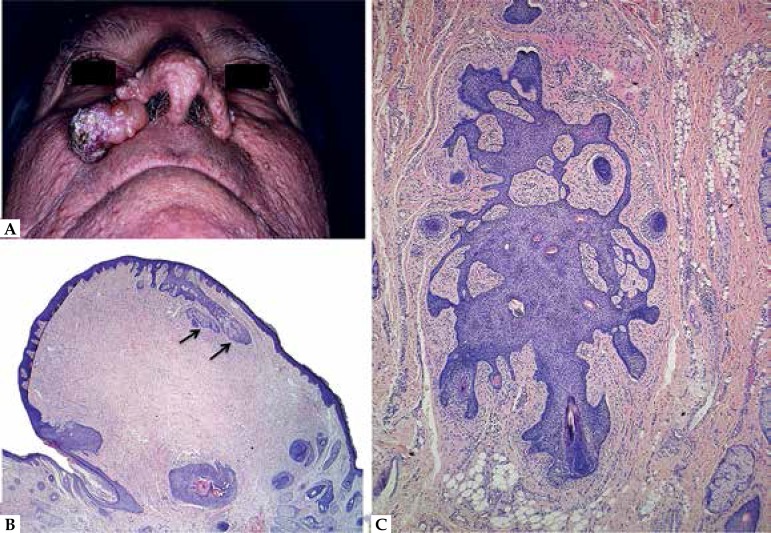
A. Birt-Hogg-Dubé syndrome. Multiple papules distributed on the face and a larger nodular lesion on the right nasal ala. B. Trichodiscoma. Papule containing a centrally located dense stroma that pushes the epithelial component to the periphery; note the small sebaceous lobules (arrows). C. Fibrofolliculoma. Vertically oriented distorted follicular structure with thin anastomosing strands surrounded by a dense stroma

Multiple fibrofolliculomas and trichodiscomas are the main cutaneous manifestations of the rare Birt-Hogg-Dubé syndrome (BHDS) (Figure 11A). In this syndrome, with autosomal dominant inheritance, there is an increased incidence of kidney neoplasms (oncocytomas, chromophobe carcinoma, papillary carcinoma, clear cell carcinoma), and lung disease (spontaneous pneumothorax, pulmonary cysts, bullous emphysema). Less often, patient may present multiple lipomas and angiolipomas, as well as neural tumors (neurothekeoma, meningeoma), connective tissue nevi, facial angiofibromas and parathyroid adenoma. The relationship with colorectal cancer is controversial. The initially reported association with medullary cancer of thyroid has not been confirmed and was instead associated with the presence of coexistent dominant hereditary multiple endocrine neoplasia type 2.^50^

The susceptibility gene for SBHD has been localized in 17p11.2 and 17p12-q11.2, and subsequently identified as a "new" supressor tumor gene designated as folliculin (FLCN). It is present in 60% to 80% of the patients

Exceptional cases of multiple trichodiscomas without any other cutaneous or extracutaneous manifestation as well as observations of localized/circumscribed SBHD have been reported.^51,52^ Fibrofolliculoma/trichodiscoma can also occur in exceptional occasions as a sporadic solitary lesion of exclusive dermatologic relevance.

**Fibrofolliculoma**: Histologic examination discloses a vertically oriented dermal papule centred by a follicular infundibulum from which anastomosing thin cellular cords, that may contain sebocytes or sebaceous lobules, irradiate into the surrounding abundant stroma (Figure 11B). Cystic dilatation of the centrally located infundibulum may occur. Stroma is readily conspicuous at low magnification, with a striking mucinous character, containing parallel fibres of fibrillary collagen, fibroblasts and occasional dendritic cells.

**Trichodiscoma**: The classic histologic picture of trichodiscoma differs from that of fibrofolliculoma by the relative proportions and disposition of the stromal and epithelial components, as well as the horizontal rather than vertical general orientation of the lesion. In trichodiscoma there is a greater development of the stroma and tendency to a more peripheral disposition of the epithelial component, which keeps its radiary disposition and tends to exhibit a more pronounced mature sebaceous content, recapitulating mantle hair differentiation (Figure 11C). The stroma, peripherally limited by the infundibulo-sebaceous cords, dominates the picture. Its nature is similar to that of fibrofolliculoma and is characteristically devoid of elastic fibres.

As seen, the distinction between fibrofolliculoma and trichodiscoma is not always possible and, due to the presence of mantle hair differentiation, the lesions are also known under the term "mantleoma".

##### Differential diagnosis:

Fibrofilliculoma/trichodiscoma has a distinctive, easily recognisable histological picture (Chart 2). Trichofoliculomas (see below) with a well-developed stroma may be confounded with fibrofolliculoma. However, in the former no radiate cords containing sebocytes emanate from the main infundibular wall, and in turn, fibrofolliculoma does not depict secondary infundibula from which vellus hair emanate.

**Chart 2:** Differential diagnosis between trichodiscoma and fibrofolliculoma

Trichodiscoma	Fibrofolliculoma
Horizontally oriented	Vertically oriented
Epithelial +	Epithelial +++
Peripheral location	Central location
Stroma +++	Stroma +++
Cystic pattern - to +	Cystic pattern ++

*Neurofollicular hamartoma*, described by Barr et al, is thought to represent a variant of fibrofilliculoma/trichodiscoma with a prominent spindle cell stroma. ^53-55^ It should be noted that neurofollicular hamartoma is a solitary papule of the nose of strict dermatologic relevance whereas fibrofolliculoma/trichodiscoma are in general multiple and a major sign of BHDS.

#### Perifollicular fibroma

Perifollicular fibroma presents as single or multiple papules most commonly located on the head and neck. Some authors have regarded it as a hamartomatous lesion and a possible variant of angiofibroma/fibrous papule, whereas others have affirmed its relation to the spectrum of fibrofolliculoma/trichodiscoma. In fact, the association of multiple perifollicular fibromas and colonic polyps is called the Hornstein-Knickenberg syndrome, which is closely related to or even part of the spectrum of Birt-Hogg-Dubé syndrome.^56,57^ In addition, lesions with overlapping features between fibrofolliculoma and perifollicular fibroma have been documented in the setting of Birt-Hogg-Dubé syndrome.^58^

Histologically, it consists of cellular fibrous tissue arranged concentrically around one or several normal hair follicles with an "onion-skin" appearance. Frequently, a cleft separates the fibrous tissue from the adjacent dermis.

##### Differential diagnosis

*Fibrofolliculoma*: the fibrous tissue surrounds and is closely intermingled with a distorted hair follicle that displays thin anastomosing projections that frequently contain sebocytes or small sebaceous lobules; in contrast, in perifollicular fibroma, the fibrous tissue surrounds normal follicular structures.

*Angiofibroma/fibrous papule*: perifollicular fibrosis tends to be less prominent, an angiomatous component is evident, and the presence of spindle-shaped or stellate fibroblasts is usually easy to identify.

#### Trichofolliculoma

The diagnosis may be clinically elicited in the presence of a smooth hemispheric papule with a central depression from which multiple thin hair emerge, located on the centrofacial area in adults.

**Figure 12:**
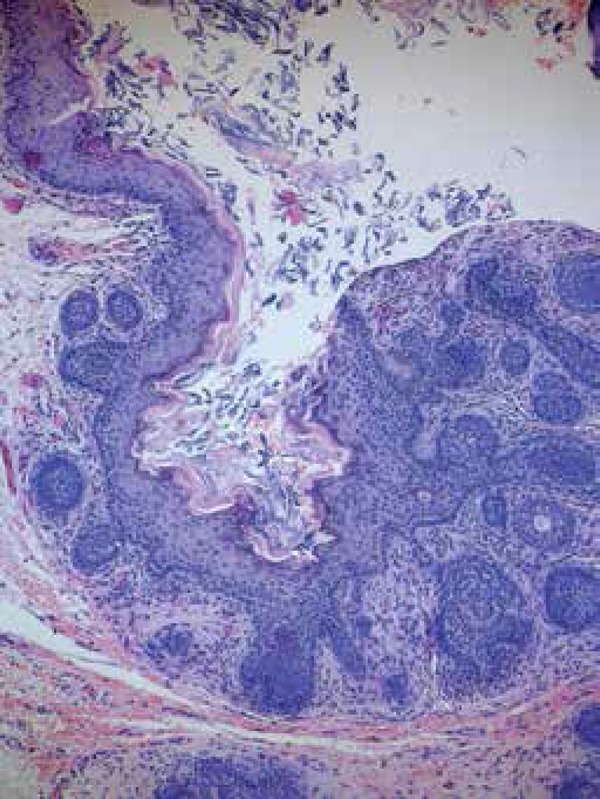
Trichofolliculoma. Cystic cavity from which numerous vellus hair follicles radiate

Histologically it corresponds to single or multiple vertically oriented infundibulocystic keratinous structure(s) draining to the skin surface by an ostium and resulting from one or multiple dilated infundibula. From their wall smaller infundibula emanate centrifugally that, in turn, give rise to mature or dystrophic vellus hair follicles (Figure 12). The follicular elements are embedded in a connective tissue, which may be poorly developed or, in contrast, abundant and fibrous, separated from the surrounding dermis by retraction clefts.^59^

##### Differential diagnosis

The distinction from pilar sheath acanthoma, dilated pore and tricoepithelioma is generally straightforward. Neither perifollicular fibroma nor fibrofolliculoma/trichodiscoma, which share a stroma similar to that of tricofolliculoma, show vellus hairs irradiating from dilated infundibulocystic structures that are characteristic of the latter.

The difference between trichofolliculoma and folliculosebaceous cystic hamartoma *(HQFS)* would rely on the operculized indundibulocystic silhouette with irradiating vellus hair follicles in the former, and the sebaceous differentiation and development of the stroma with obligatory mesenchymal changes (adipocytes) in the latter. However, the fact that both can share the same architectural pattern, the possibility of sebaceous differentiation in trichofolliculoma ("sebaceous tricofoliculoma") and the report of cases where distinction between these tumors is virtually impossible, may indicate a close relationship between these lesions and/or a common pathogenic basis.^60^

#### Folliculosebaceous cystic hamartoma

Clinically it is a slow growing solitary nodule of the head and neck, as a rule first noticed in the 5th or 6th decades. Congenital forms are not exceptional. Lesion can attain a large size and be cerebriform.

The histological characterization of the folliculosebaceous cystic hamartoma has been thoroughly precised in the initial description: a dermal infundibulocystic structure into which merge sebaceous ducts attached to sebaceous lobules, surrounded by a fibroplasic lamellar stroma that incorporates mesenchymal elements, including adipocytes, separated from the surrounding dermis by clefting. ^61^ The individualization of folliculosebaceous cystic hamartoma as an entity distinct from trichofolliculoma is controversial. The prominence and character of the stroma as well as the non-operculized general disposition and magnitude of the sebaceous differentiation would assist the diagnosis of folliculosebaceous cystic hamartoma rather than sebaceous trichofolliculoma (see above)

It should be noted that folliculosebaceous cystic hamartoma bears no relationship with Torre-Muir syndrome.

## Questions

**1. Which one of the following is correct about follicular tumors?**
- Most cases are clinically distinctive.
- They occur more commonly as multiple lesions.
- The diagnosis is based on histology.
- They occur more commonly on the limbs.

**2. Which one of the following is not characteristic of outer root sheath differentiation?**
- Basket-weave orthokeratosis.
- Cells with pale cytoplasm.
- Peripheral palisading.
- Thickened hyaline basement membrane.

**3. Which one of the following does not belong to the group of tumors with germinative (hair germ) differentiation?**
- Trichoblastoma.
- Pilomatricoma.
- Trichogerminoma.
- Tricoepithelioma.

**4. Which one of the following tumors, when multiple, can be associated with increased risk of visceral neoplasms?**
- Pilar sheath acanthoma.
- Dilated pore of Winer.
- Proliferating trichilemmal tumor.
- Trichilemmoma.

**5. Which one of the following is not a feature of Birt-Hogg-Dubé syndrome?**
- Multiple tumors of follicular infundibulum.
- Multiple fibrofolliculomas and trichodiscomas.
- Increased risk of renal cancer.
- Spontaneous pneumothorax.

**6. Which one of the following is true about trichoblastoma?**
- The histological differential diagnosis with basal cell carcinoma is straightforward, even in small biopsies.
- Artifactual clefts are typically seen between the epithelial nests and the stroma.
- The presence of CK20-positive cells is consistent with the diagnosis of trichoblastoma.
- It is a rare occurrence in organoid nevus (nevus sebaceous).

**7. Which one of the following syndromes is not usually associated with multiple pilomatricomas?**
- Myotonic dystrophy.
- Rombo syndrome.
- Gardner syndrome.
- Rubinstein-Taybi syndrome.

**8. Which gene is associated with Birt-Hogg-Dubé syndrome?**
- PTEN.
- CYLD.
- FLCN.
- WNT.

**9. Which one of the following is not usually included in the histological differential diagnosis of desmoplastic trichoepithelioma?**
- Infiltrative basal cell carcinoma.
- Squamous cell carcinoma.
- Microcystic adnexal carcinoma.
- Syringoma.

**10. Which one is not usually found in pilomatricoma?**
- Foreign body giant cell reaction.
- Ghost cells.
- Mitotic activity.
- High-grade nuclear atypia.

**11. Which one is true about proliferating trichilemmal tumor/cyst?**
- It is more common in men.
- It is usually multiple.
- The upper trunk is the most common location.
- The main differential diagnosis is squamous cell carcinoma.

**12. Which one is false about trichogerminoma?**
- It belongs to the group of tumors with germinative (hair germ) differentiation.
- It usually presents with multiple lesions.
- It is more common on the head/neck.
- The characteristic histological finding is the presence of “cell balls”.

**13. Regarding the tumor of follicular infundibulum (infundibuloma), which one is false?**
- In its multiple form (infundibulomatosis) the lesions can be confused with pityriasis versicolor, vitiligo or porokeratosis.
- In its multiple form (infundibulomatosis) there is a well-documented association with visceral malignancies, particularly of the thyroid and breast.
- When single, it is usually not clinically distinctive and can be confused with a seborrheic keratosis or a basal cell carcinoma.
- Histologically, it is a subepidermal plaque-like proliferation of pale keratinocytes that may display peripheral palisading and stromal condensation of elastic fibers.

**14. Which one is false about trichofolliculoma?**
- It occurs more commonly on the head/neck.
- A typical presentation is a papule with protruding hairs.
- The distinction from pilar sheath acanthoma is usually not problematic.
- The distinction between sebaceous trichofolliculoma and folliculosebaceous cystic hamartoma is straightforward.

**15. Which one is false about adamantinoid trichoblastoma?**
- It is also known as cutaneous lymphadenoma.
- It is typically seen in the pediatric population.
- It is usually well-circumscribed.
- The nests of cells are infiltrated by lymphocytes and dendritic cells.

**16. Which one of the following favours clear cell poroma over trichilemmoma?**
- Peripheral palisading.
- Hyaline basement membrane.
- Folliculocentricity.
- Ductal differentiation.

**17. Which one of the following features in not seen in trichilemmoma?**
- Lobular architecture.
- PAS-positive cells.
- Ghost cells.
- Cytopathogenic effect of HPV.

**18. Which one of the following proteins is thought to have a pathogenic role in pilomatricoma?**
- CD34.
- Beta-catenin.
- CK20.
- EMA.

**19. Which tumor is seen both in Rombo and Bazex- Dupré-Christol syndromes?**
- Trichilemmoma.
- Trichoepithelioma.
- Trichoepithelioma.
- Pilar sheath acanthoma.

**20. In addition to tricoepitheliomas, mutations in the CYLD gene can be associated with which combination of adnexal neoplasms?**
- Sebaceomas and sebaceous adenomas.
- Syringomas and hidradenomas.
- Spiradenomas and cylindromas.
- Trichilemmomas and tumors of follicular infundibulum.

**Table:**

**Answer key**			
Coccidioidomycosis and the skin: a comprehensive review. 2015;90(5):610-21.			
1. C	6. C	11. D	16. A
2. B	7. A	12. B	17. D
3. A	8. D	13. D	18. B
4. B	9. D	14. B	19. A
5. B	10. C	15. C	20. A

Papers Information for all members: The EMC-D questionnaire is now available at the homepage of the Brazilian Annals of Dermatology: www.anaisdedermatologia.org.br. The deadline for completing the questionnaire is 30 days from the date of online publication.
